# The light-oxygen effect in biological cells enhanced by highly localized surface plasmon-polaritons

**DOI:** 10.1038/s41598-019-54905-5

**Published:** 2019-12-05

**Authors:** Anna Khokhlova, Igor Zolotovskii, Sergei Sokolovski, Yury Saenko, Edik Rafailov, Dmitrii Stoliarov, Evgenia Pogodina, Vyacheslav Svetukhin, Vladimir Sibirny, Andrei Fotiadi

**Affiliations:** 1 S. P. Kapitsa Technological Research Institute, Ulyanovsk State University, Ulyanovsk, 432017 Russia; 2 Aston Institute of Photonic Technologies, Aston University, Birmingham, B4 7ET UK; 3 International Center of Critical Technologies in Medicine, Saratov State University, Saratov, 410012 Russia; 4 Scientific-Manufacturing Complex (SMC) “Technological Centre”, Zelenograd, Moscow 124498 Russia; 5 Department of Bioenergetics and Food Analysis, Faculty of Biology and Agriculture, University of Rzeszow, Ćwiklińskiej St., Rzeszów, 35-601 Poland; 6 Electromagnetism and Telecommunication Department, University of Mons, Mons, 7000 Belgium

**Keywords:** Biological physics, Biophotonics, Lasers, LEDs and light sources, Biophysics, Micro-optics

## Abstract

Here at the first time we suggested that the surface plasmon-polariton phenomenon which it is well described in metallic nanostructures could also be used for explanation of the unexpectedly strong oxidative effects of the low-intensity laser irradiation in living matters (cells, tissues, organism). We demonstrated that the narrow-band laser emitting at 1265 nm could generate significant amount of the reactive oxygen species (ROS) in both HCT116 and CHO-K1 cell cultures. Such cellular ROS effects could be explained through the generation of highly localized plasmon-polaritons on the surface of mitochondrial crista. Our experimental conditions, the low-intensity irradiation, the narrow spectrum band (<4 *nm*) of the laser and comparably small size bio-structures (~10 μm) were shown to be sufficient for the plasmon-polariton generation and strong laser field confinement enabling the oxidative stress observed.

## Introduction

These days lasers are widely used in treatment of neoplastic diseases^1–3^, for photomodulation of cellular processes *in vitro* and *in vivo*^4^. The different laser techniques involved solely and in combination with photodynamic, chemotherapeutic and radiotherapy are widely used in cancer therapy. The intensive laser irradiation applied directly to the tissue causes the direct thermos ablation of surface cancers like cell carcinoma^5^. However, it can cause serious drawbacks associated with necrotic damage of the tissue below the treated tumor tissue. Photodynamic therapy is most well developed and used in oncology optical treatment where the lasers are widely employed to induce the photo damage of the cancer cells previously uploaded with photosensitizer. With all advantages of this approach, non-invasiveness, precisive control of the irradiation dose it has significant disadvantages, i.e. intrinsic and photo- toxicity of the photosensitizers. Another approach where light irradiation is widely used at comparably low intensities is photobiomodulation (PBM) which is very promising laser therapy that is based on the laser-induced intracellular ROS generation^6–9^.

The wide use of the PBM therapy (PBMT) in the biomedical and related areas is due to several advantages it has over ablative and photodynamic cancer therapy: non-invasiveness, high selectivity without toxicity of the photosensitizers as for PDT, high precise dose control, short treatment duration, re-treatment without risk of complications. For instance, during the PDT photosensitizers can provoke undesirable photodermatitis or even burnings, as they are absorbed by both cancerous and skin epidermal tissues^10,11^. Besides, the concentration of photosensitizers in tumor tissues is rather low, and the lasers used are not able to penetrate the tissue deeply enough, thereby weaken the photodynamic effect.

Since PBMT can be used without photosensitizers^12–15^ based on the light-oxygen effect (direct generation of singlet oxygen by laser radiation without the use of sensitizers) it is of a great practical importance in the medicine and the cell free radical biology. Although the LO effect has reliably been demonstrated in several research works, underlying fundamental mechanism of the LOEs is still unclear^8,14,16,17^.

Molecular oxygen in a triplet ground state O_2_ (^3^Σg) could be excited to singlet levels of O_2_ (^1^Δg) and O_2_ (^1^Σg)^18,19^. These electronic states differ by the spin and population of degenerated electron orbitals. The O_2_ (^1^Δg) state is degenerated then. The O_2_ (^1^Σg) state possesses a short lifetime of about 10 picoseconds and relaxes fast to the lower-energy excited state O_2_ (^1^Δg). The O_2_ (^1^Δg) state is referred to as singlet oxygen (SO).

The energy gap between the ground state and singlet oxygen is 0.98 eV per molecule. It is associated with the optical transition in the near IR range at the wavelength of about 1268 nm. Moreover, for an isolated molecule such direct transition is forbidden by the selection rules due to spin and orbital symmetry^20,21^. This sets a very small probability of singlet oxygen generation without photosensitizers. The peak absorption cross-section for the transition *O*_2_(^3^Σ_*g*_) → *O*_2_(^1^Δ_*g*_) in water around 1260–1270 nm measured in^13,22,23^ are *σ*_*ox*_ ≃ 2,4 ∗ 10^−23^ *cm*^2^ and *σ*_*ox*_ ≃ 7,4 ∗ 10^−24^ *cm*^2^, respectively. These values are at least 5 orders of magnitude lower than the absorption cross-section in the near-IR absorption band (including 1260–1270 nm) reported a typical photosensitizer (e.g. porphyrins), *σ*_*ss*_ ≈ 10^−18^–10^−19^ *cm*^2^ ^24^.

With the use of photosensitizers, the typical CW laser intensities used for effective singlet oxygen generation are *I* ~ 100 *mW*/*cm*^2^ ^13,25–27^. Therefore, for similar effects in organic media without photosensitizers could expect the threshold laser intensities higher than *I* ~ *I*_*ss*_*σ*_*ss*_/*σ*_*ox*_ ≫ 10^2^ *W*/*cm*^2^. Such estimations are in agreement with the results of experiments^9^.

On the other hand, the laser radiation of such high intensities should immediately cause thermal destruction of any organic material. Remarkably, in our experiments the cell oxidative stress (associated with the singlet oxygen generation) has been undoubtfully observed with only *I* ~ 100 *mW*/*cm*^2^ laser irradiation at 1265–1268 *nm*, i.e. at the intensity typically used in PBMT^8,28–33^.

**Figure 1:**
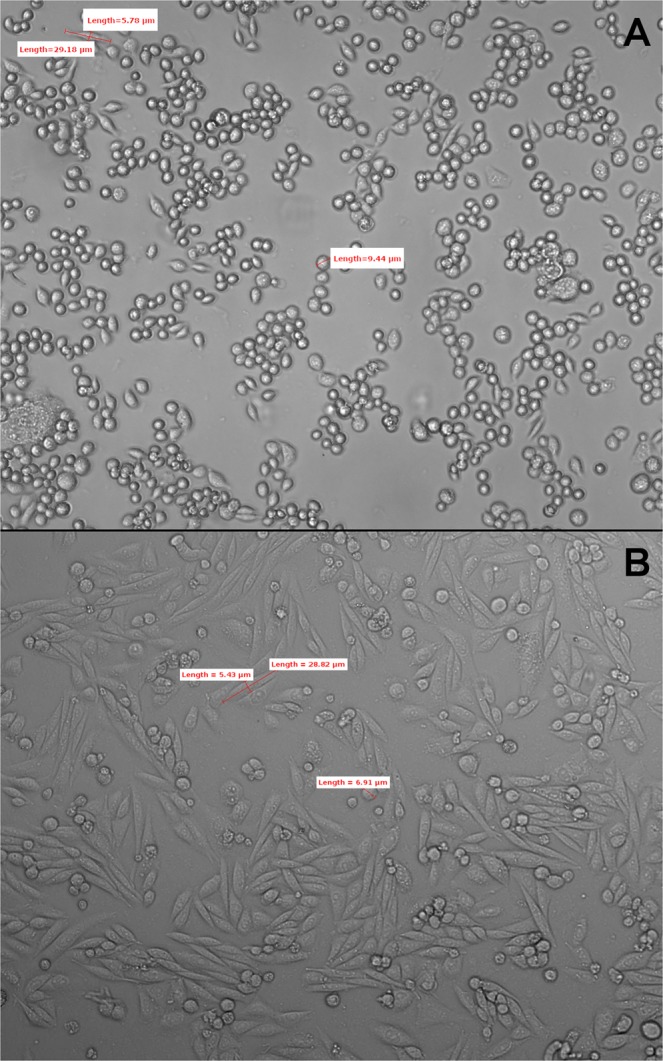
(**A**) HCT116 cell culture before the experiment. (**B**) CHO-K1 cell culture before the experiment. LM ×100.

In this work, we offer a potential mechanism which can be responsible for the light-oxygen effect induced in cells by the low-intensity laser radiation. In our experiments, the isolated cells (separately located (Fig. 1)) illuminated with a narrow-band CW (continuous wave) laser of average power as low as 2 mW and linewidth less than 0.1 pm tunability over the spectrum range revealed a strong resonance dependence of the ROS concentration on the laser wavelength. In order to explain the observed effect, we proposed a model that involves surface plasmon polaritons which are presumably generated on the surface of the inner mitochondrial membrane.

Such strong light field confinement achieved through plasmon-polariton generation is currently known for ordered metallic nanostructures irradiated by narrow-band lasers^34,35^. They are characterized by a very narrow resonant acceptance band^35^ mainly determined by the structure design (single- and multi-antenna structures)^34^, where enhancement of a local field intensity up to 4 orders of magnitude^36–47^ could be achieved.

Similar to metallic nanostructures we demonstrate here that plasmon-polariton waves on the surface of the inner mitochondrial membrane could be generated at extremely narrow wavelengths and provide a strong optical field confinement inside the cells finally enabling ROS generation bringing the cells to the significant oxidative stress. In contrast to previous research on the cell oxidative stress^13,17^, where laser sources with linewidth of ~1 nm and broader (typically, 4–6 nm) were used, in our experiments we employed a low-intensity tunable laser with a linewidth of Δ*λ* < 1 *pm* that was commonly used for effective excitation of plasmon polaritons in metallic nanostructures^48^). The plasmon-polariton model of low-level laser irradiation (LLL) effects is not in contradiction with the previously proposed models. In particular, the non-specific pathway mediated response of light describes non-resonant interaction of LLL with cells^49^. In accordance with that model, the LLL effect is amplified through the coherent interaction, where the absorbing molecules are assumed to be identical and cooperating with each other to form the coherent states. In our experiment, the irradiated cells are spatially separated and have different metrics (~10 µm) (Fig. 1). In principle, we can accept that the absorbing molecules form a coherent state within one separated cell. However, the size of such coherent domain is too small to match so narrow spectrum peak as observed in the experiments. To explain the narrow peak width, a different mechanism enabling cooperation between molecules relating to different separate cells should be suggested. Existence of such a mechanism is rather questionable. Plasmon-polariton wave generation proposed in the paper is an alternative way to explain the enhancement of the LLL-induced cellular ROS effects.

## Experiment

Under irradiation in the visible range, the ROS are generated in the mitochondria^50^. Main aim of this research was to measure the intracellular ROS formation after the narrow-band irradiation using the laser tuned within the spectrum range of ~1260–1270 nm. The mitochondria due to its unique highest membrane structure packing per volume are ideal object for such study. This is due to the presence of cristae, the mitochondrial inner membrane structure, ranging in size from 100 to 500 nm^51–53^. Two cell types, a human cancer and non-cancerous animal, were chosen for our experiments, correspondently human colorectal cancer cell line HCT116 (ATCC CCL-247TM, Manassas, VA, USA) and the Chinese hamster ovary cell line CHO-K1 cells (ATCC CCL-61TM, Manassas, VA, USA). Both cell cultures were maintained in RPMI-1640 medium (Paneco, Russia), supplemented with 10% fetal bovine serum and gentamycin at a final concentration of 50 µg/ml. Twenty four hours before the experiment the cells were transferred in the slide chamber (SPL LifeSciences, Korea) at a concentration of 10^5^ cells/ml. Figure 1 shows the HCT116 (A) and CHO-K1 (B) cells a day after a passage to the slide chamber immediately before the experiment (experiment No. 1). The diameter of a single cell is about 10 and 7 µm for the HCT-116 and CHO-K1 cell lines, respectively. Four separate assays were performed with cells of the same type and more than 300 cells were analyzed in each experiment.

After cells adhered to the slide chamber bottom and reached the early exponential phase of the growth, the cell cultures were irradiated for 30 min by a semiconductor highly coherent laser with a linewidth less than 0.1 pm (Yenista Optics, OSICS T100 Tunable Laser Module T100 1310 (France)) tunable over the range of 1260–1360 nm. All irradiation procedures were performed in the outer incubator chamber (UNO, OkoLab, Italy) at 100% humidity, 37 °C, and 5% CO_2_. All ROS concentration measurements were performed under the same experimental conditions at different laser wavelengths 1260, 1263, 1265, 1268 and 1270 nm. Four experimental series were carried out for each laser wavelength. The laser power and the irradiation intensity were ~2 mW and 2 mW/cm^2^, respectively. During the assay, unirradiated cells shielded by the aluminum foil were used as a control in the same slide chamber. A series of preliminary measurements of the temperature in the culture medium incubated at 37 °C in the slide chamber revealed no temperature increases during the laser irradiation.

Intracellular ROS concentration was measured immediately after the laser exposure. For this purpose, the growth medium was removed from the experimental and control cells and a mixture of 1× sodium phosphate buffer (PBS) (pH 7.4) and 2′,7′-dichlorohydrofluorescein diacetate fluorescent dye (DCF-DA, Sigma-Aldrich, USA) was added at a final concentration of 30 μM. The dye was loaded for 20 minutes in a CO_2_ incubator at 37 °C. Then a mixture of PBS and dye was removed from the cell, the cells were washed in pure PBS for 10 minutes at 4 °C.

The fluorescence images of the cells were captured using an optical system comprising Nikon Тi-S microscope, DS-Qi1MC camera, Nikon S Plan Fluor ELWD 20 × 0.45 lens and appropriate filter and PC with NIS-elements 4.0 package. The measured integral cell fluorescence relating to the noise level was used to estimate the intracellular ROS concentration^54^. Then we determined the ratio between the fluorescence relating to irradiated and control cells. The ImageJ v. 1.48 software was used for image processing. Statistical data were processed in Microsoft Excel using Student t-test criteria for paired variables with p < 0.05.

**Figure 2:**
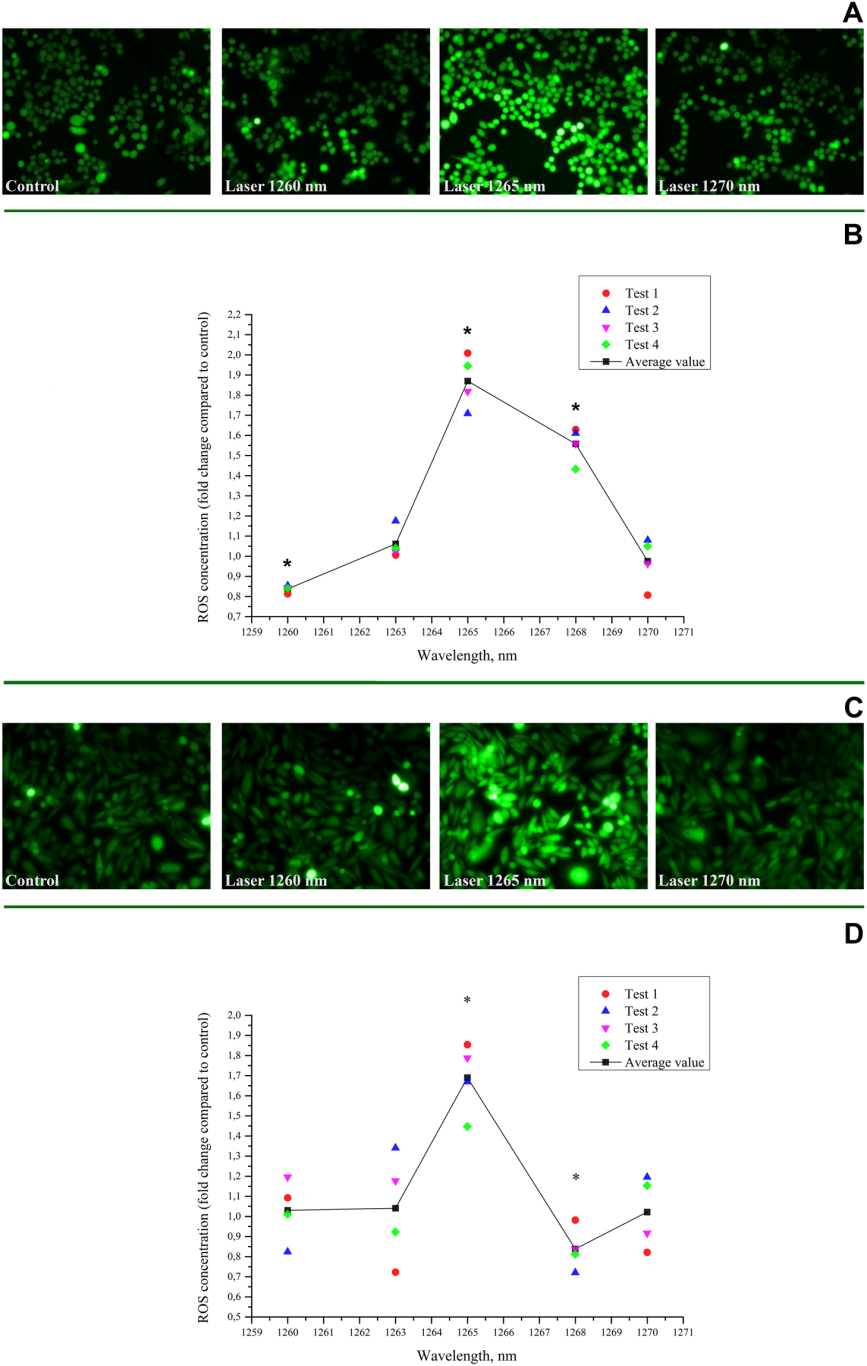
(**A**) The images of the fluorescence of the HCT116 cells stained with DCFH-DA: unirradiated (control) and exposed to the laser irradiation at 1260, 1265, 1270 nm. (**B**) Active spectrum of the laser irradiation of 1260–1270 nm effect on the intracellular ROS concentration in HCT116 cells after. (**C**) The microscopic pictures of the fluorescence of the CHO-K1 cells stained with DCFH-DA: unirradiated (control) and exposed to the laser irradiation at 1260, 1265, 1270 nm. (**D**) Active spectrum of the laser irradiation (1260–1270 nm) effects on the intracellular ROS concentration in CHO-K1 cells. *Statistically significant differences between control and laser irradiated cells (p < 0.05).

The results of the experiments are shown in Fig. 2. One can see that the level of oxidative stress inside the cell induced by low-intensity laser irradiation exhibits a strong resonant dependence on the laser wavelength demonstrating the peak response at the 1265 nm.

## Discussion

There are two important experimental observations that require an advanced explanation. First, the cells had ROS response induced by very low laser intensity and that was in contradiction with a low absorption cross-section measured with the organic materials^2,55,56^. Second, the very narrow spectral band where the laser-induced ROS effect was most prominent is also in contradiction with a relatively small size of the cells. Indeed, the spectral width of the resonance curve shown in Fig. 2 (where the anomalously high generation of the ROS was detected) is Δ*λ* ~ 4 *nm* only or Δ*ω* ~ 410^12^ *s*^−1^. The light coherence length *l*_*C*_ = *πc*/Δ*ω*^57,58^ corresponding to this bandwidth is estimated as ~0.25 *mm*. The typical linear size of irradiated colonies shown in Fig. 1 is *l*_*D*_ ~ 10 *μm*. Since the condition *L*_*c*_/*n*_*eff*_ < *l*_*D*_ must be satisfied for any transparent non resonance media, i.e. the coherence length determining the absorption linewidth (see Fig. 2) must be smaller than the length of irradiated singly located cells, and *l*_*C*_/*l*_*D*_ ~ 25, it may be concluded that the effective refractive index *n*_*eff*_ > 25 is formed in the irradiated cell. In other words, the speed of light in the irradiated cell is *V* = *c*/*n*_*eff*_ ≤ 1, 2 ∗ 10^7^ *m*/*s*. A well-known physical mechanism enabling such a strong slowing down of light is the plasmon-polariton interaction in metal films^59–62^.

The surface plasmon polariton (SPP) is the bound state of oscillating electrons with the electromagnetic field of laser radiation at the boundary of a conducting medium. The SPPs can be generated when a conductive film and a structure (diffraction gratings, prisms) are combined. The structure provides phase matching between the external radiation and the SPP generated on the film surface. The inner membrane of the mitochondria can play the same role inside of the eukaryotic cell. Indeed, the inner membrane could be taken as a diffraction grating covered by a chain of metal-containing proteins that can play a role of a conductive film. During the respiratory functioning the mitochondrion continuously generates nonequilibrium charges at the surface of the protein “film” fulfilling the ideal conditions for SPPs formation under the laser irradiation.

These phenomena may then help to explain the low-intensity light-oxygen effect generated in individual cells with narrow linewidth laser irradiation observed. In particular, such consideration could appear to be in fact the generation of highly localized ultra-slow surface plasmon polaritons (SPP) on the mitochondria inner membrane structure. The detectable increase in intracellular ROS concentration under low-intensity laser radiation (below 1 *W/cm*^2^) presumes the possibility of the SPPs (with significant local field amplification) in mitochondria. The inner mitochondrial membranes being the location of the protein complexes composing the respiratory chain are the main source of ROS generating in the living cell.

Since the electron transport chain of the respiratory enzymes is localized on the inner mitochondrial membrane and the proton pumps provide a voltage gradient for positively charged hydrogen ions on its surface, we can suggest that areas of nonequilibrium charges are formed inside the inner membrane^63^.

Since, the electron transport chain provides cascade of the electron transferred between metal atoms in the protein complexes of the mitochondrial respiratory chain it is highly likely that the SPPs are formed in these areas. It is well known that the cytochrome *c* and ubiquinone molecules, carriers of the free electrons in electron transport chain (ETC) could support the formation of the nonequilibrium charges in these areas^63,64^. Brazhe *et al*.^64^ uncovers that the plasmon resonance is induced in the mitochondrial cristae inner membrane. Therefore we assume that the similar plasmon resonance can spontaneously appear in the mitochondria due to the formation of the nonequilibrium chargers there.

Since the current (electron flow) passes through mitochondrial membrane (parallel to its surface) it can be considered as a conductor^63^. The dispersion relation determining the SPP wave number *β* for such structure can be expressed as^47,59,60,65–68^

1 $$\exp \,(\,-\,2{q}_{2}h)=\frac{{q}_{2}{\varepsilon }_{1}+{q}_{1}{\varepsilon }_{2}}{{q}_{2}{\varepsilon }_{1}-{q}_{1}{\varepsilon }_{2}}\cdot \frac{{q}_{2}{\varepsilon }_{3}+{q}_{3}{\varepsilon }_{2}}{{q}_{2}{\varepsilon }_{3}-{q}_{3}{\varepsilon }_{2}},$$

where, ${q}_{j}=\sqrt{{\beta }^{2}-{k}_{0}^{2}{\varepsilon }_{j}}$, *j* = 1, 2, 3, *k*_0_ = *ω*/*c* and *c* is the wave number and speed of light in vacuum, respectively; *h* is the thickness of the conductive film (the thickness of inner mitochondrial membrane, i.e. *h* ~ 2 *nm*), *ε*_*i*_ is the dielectric permeabilities of the surrounding medium *ε*_1,3_ and film *ε*_2_, respectively, β is the SPP wave number.

The problem of a dielectric permeability in biological objects is quite complex question. However, in the case of the thin film (i.e. for *λ* ≪ *h*), in terms of Eq. (1) the expression for the SPP wave number can be written with high accuracy as^65–68^

2 $$\beta \sim 1/h,$$

where *h* is the mitochondrial membrane thickness (conductive film). In the case of a typical mitochondrial membrane, *h* ~ 2 *nm*. Thus, the effective refractive index for a film can be defined as

3 $${n}_{eff}\sim \lambda /h\gg {10}^{2}.$$

Self-consistent operation of the proposed mechanism requires phase-matching of the generated ultra-low SPPs on the surface of mitochondrial membranes. Ultra-slow SPPs are known to be excided in thin-film structures due to use of diffraction gratings^47,59–62^. In this case,

4 $$\beta (\omega )\equiv {n}_{eff}\omega /c\approx 2\pi /{\Lambda }_{eff},$$

where Λ_*eff*_ is some period of the conductive film.

A lipid bilayer or periodically spaced mitochondrial cristae forming kind of dimensional periodic structures^69^ could operate as an analogue of diffraction gratings in the thin films. In addition, the quasi-periodic structure in the considered films can be obtained from specially arranged membrane proteins^63^. The described plasmon resonance enables a local more than three orders of magnitude increase in the field intensity on the surface of nanoscale mitochondrial membranes. Such localization believed can enable direct excitation of singlet oxygen without photosensitizers.

We have to admit that strict two-dimensional periodicity of lipid arrays assumed in Eq. 4 is an idealization enabling the maximal optical field localization on the membrane surface. In reality, geometrical distribution of the lipids in an array possesses a limited regularity with some dominating period Λ_0_ that just makes the reported effect less pronounced. Besides, some optical field enhancement is achievable at Λ_*eff*_ = Λ_0_*m*, where *m* is an integer. The typical lipid structure period is estimated to be ~1 nm, enabling Λ_*eff*_ for optical field enhancement to be 1–10 nm.

It is important to stress that similar laser irradiation effects were obtained on the different cell lines (non- vs cancerous, and non- vs human ones). This could be explained by the fact that the inner mitochondria membranes of eukaryotic cells have almost identical physical characteristics (thickness, periodicity, arrangement of bilipid layers, etc.). Therefore, the similarity of the laser effects observed on the different types of cell cultures could be considered as strong evidence of the mitochondrial origin of the light-oxygen effect appearing in the living cells under the narrow band near-infrared irradiation.

Concluding, the presented results are strongly braced by data where the lasers with different spectrum widths differently affected the cell functionalities. These lasers were low-power high-coherent (narrow-band) one with a high spectral density of radiation and wide-band one with a relatively large lasing line width. The same HCT116 colorectal cancer cells (ATCC CCL-247^TM^, the American Type Culture Collection, USA) were irradiated with linewidths of low and medium power lasers of the wavelength of 1265 nm. In this case, the energy densities were 9.45 (narrow coherent, low-power laser) and 66.6–400 J/cm^2^ (wide-band, medium-power laser), respectively. With the same exposure time the results of the experiments allow us to conclude that both a narrow-band irradiation source and a wide-band laser at the appropriate wavelength both caused oxidative stress, leading to the death of cancer cells and can disrupt the work of the mitochondria at each energy density used. At the same time, the pronounce^a^^1^ of the corresponding processes (associated with the development of oxidative stress) was quite comparable, while the intensity of radiation from the corresponding sources differed by more than an order of magnitude. The explanation for this “paradox” might be in the spectral brightness of a low-power narrow-band source which is comparable and even exceeds the spectral brightness of a wide-band fiber laser. Thus, the results presented in^70^ then also indicate the formation of the surface plasmon polaritons of resonance nature in cells with narrowband 1265 nm laser.

## Conclusion

In this work we reveal the singlet oxygen generation induced by the laser radiation in individual CHO-K1 and HCT116 cells (with the size of about 7–10 µm), which demonstrated narrow-band resonant sensitivity at 1265 nm. Therefore, we assume that the observed narrow band resonance could be explained through the generation of the localized slow waves plasmon resonance in the cells characterized by a high effective refractive index (>25). The mitochondrial membrane surfaces being highly electrically conductive are the most probable object, where such plasmon polaritons can be generated. The conditions for phase-synchronization could be founded in the periodic organic structures such as the lipid bilayer or mitochondrial cristae. In this context, the observed light-oxygen effect of low-intensity laser irradiation can be explained by the formation of highly localized plasmon-polariton wave packs which enable the generation of singlet-oxygen in the strong field localization points. This plasmon-polariton explanation of the LLL-induced cellular ROS effect is in agreement with the reported experimental data. Besides, in our recent experiments we have directly compared the effects induced by a low-intensity narrowband (highly coherent) and relatively high-intensity wideband lasers exploring specific features of two processes^70^. These new observations are also consistent with plasmon-polariton interpretation of the effect proposed here.

## Footnotes

1 aIn this case it means that the irradiation with different lasers (low-power narrow-band and broad-band medium power) can generate nearly the same amount (per mass unit) of the ROS, while the radiation doses in both cases differ by more than order. The reasonable explanation of this phenomenon may be that not dose itself but also the spectral density is important in generating the same ROS amounts for both laser types. Therefore, it is clear that the revealed cellular effects have of a pronounced resonant nature.
