# Author Correction: The light-oxygen effect in biological cells enhanced by highly localized surface plasmon-polaritons

**DOI:** 10.1038/s41598-020-58159-4

**Published:** 2020-01-22

**Authors:** Anna Khokhlova, Igor Zolotovskii, Sergei Sokolovski, Yury Saenko, Edik Rafailov, Dmitrii Stoliarov, Evgenia Pogodina, Vyacheslav Svetukhin, Vladimir Sibirny, Andrei Fotiadi

**Affiliations:** 1 S. P. Kapitsa Technological Research Institute, Ulyanovsk State University, Ulyanovsk, 432017 Russia; 2 Aston Institute of Photonic Technologies, Aston University, Birmingham, B4 7ET UK; 3 International Center of Critical Technologies in Medicine, Saratov State University, Saratov, 410012 Russia; 4 Scientific-Manufacturing Complex (SMC) “Technological Centre”, Zelenograd, Moscow 124498 Russia; 5 Department of Bioenergetics and Food Analysis, Faculty of Biology and Agriculture, University of Rzeszow, Ćwiklińskiej St., Rzeszów, 35-601 Poland; 6 Electromagnetism and Telecommunication Department, University of Mons, Mons, 7000 Belgium

Correction to: *Scientific Reports* 10.1038/s41598-019-54905-5, published online 05 December 2019

This Article contains a typographical error in the Acknowledgements section.

“Ministry of Education and Science of Russian Federation (Grant № 12.1223.2017/AP to S.S. and E.R.).”

should read:

“Ministry of Education and Science of Russian Federation (Grant No. 18-15-00172 to S.S.and E.R.)”

